# *Ptilagrostis contracta* (Stipeae, Poaceae), a New Species Endemic to Qinghai-Tibet Plateau

**DOI:** 10.1371/journal.pone.0166603

**Published:** 2017-01-06

**Authors:** Zhong-Shuai Zhang, Ling-Lu Li, Wen-Li Chen

**Affiliations:** 1 State Key Laboratory of Systematic and Evolutionary Botany, Institute of Botany, Chinese Academy of Sciences, Beijing, China; 2 University of Chinese Academy of Sciences, Beijing, China; Institute of Genetics and Developmental Biology Chinese Academy of Sciences, CHINA

## Abstract

A new species, *Ptilagrostis contracta*, endemic to Qinghai-Tibet Plateau is described and illustrated. It is distinguished from other species in *Ptilagrostis* by having contracted panicles, 1-geniculate awns with hairy columns and scabrous bristles and evenly pubescent lemmas. Evidence from lemma epidermal pattern, cytology and molecular phylogenetic analyses based on the nuclear ITS sequence data confirm its systematic position in *Ptilagrostis*.

## Introduction

*Ptilagrostis* Griseb. is a small genus of approximately 11 high altitude species, widely accepted on the basis of its distinctive morphological characters, cytological evidence [1–6] and molecular data [7, 8]. *Ptilagrostis* is disjunctive in Asia and North America [5, 9–12] with the diversity center in the Qinghai-Tibet Plateau, where approximately eight taxa occur. They grow in various alpine habitats, including meadows, grasslands, shrubby and even *Abies* forests.

During several botanical expeditions to Sichuan Province in 2013–2015, some unusual populations of *Ptilagrostis* were discovered in Litang and Kangding counties, located in the southeastern Qinghai-Tibet Plateau. The plants can be distinguished from all the other known *Ptilagrostis* species by having contracted panicles, 1-geniculate awns with pubescent columns and scabrid bristles and evenly pubescent lemmas. We determined that these populations represent a hitherto undescribed species and named as *P*. *contracta*. It is strongly supported to be a member within *Ptilagrostis* by the evidence from lemma epidermal pattern, cytology and molecular phylogenetic analyses based on the nuclear ITS sequence data. The detailed description of the new species is given below.

## Material and Methods

### Ethics statements

All the collecting locations of the new species reported in this study are not in any natural conservation area and no specific permissions were required for these locations. And our field studies did not involve any endangered or protected species.

### Material collection

A total of five populations of new species were collected and examined during the field work conducted in the Qinghai-Tibet Plateau from 2013 to 2015. The specimens from PE, HNWP, KUN, CDBI were carefully checked, as well as the specimens borrowed from HITBC, B, CAS, GOET and K.

### Chromosome preparations

Matured caryopses and voucher specimen were collected by the authors during the field trip in Litang. Voucher specimen: CHINA. Sichuan, Litang, Long. 100.41842 E, Lat. 29.72025 N, 3701 m, 26 September 2014, *Z*. *S*. *Zhang & L*. *L*. *Li 341*, deposited in the Herbarium, Institute of Botany, CAS (PE).

Vigorous root tips obtained from germinated caryopses were pre-treated in an ice water mixture at 0° for 24 hours, then fixed in Carnoy’s fluid (3: 1 ethanol: glacial acetic acid) at 4° for at least 30 min. They were then digested at 37° in a combination (1:1) of 2.5% cellulase and 1.25% pectinase for 1 hour before being stained with improved carbol-fuchsin solution [13] and squashed for cytological observation. Permanent slides were made using the standard liquid nitrogen method. The photographs were taken using a Zeiss Axio Imager A1 camera. The karyotype was analyzed following Li and Chen [14].

### Lemma epidermal pattern

Lemma ultra structure was studied by using dry mature caryopses sampled from the type specimen. To remove epicuticular wax the lemmas were cleaned in xylene for an hour. Samples were mounted and then covered with gold from a vacuum spray gun (type S-800, HITACHI). All the observation and photographs were made at varying magnifications by using scanning electron microscope (Hitachi S-4800 FESEM, Hitachi, Tokyo, Japan).

### Molecular systematics

#### Taxon sampling

In order to determine the systematic position of the new species, one new ITS sequence, for *Ptilagrostis contracta*, was added to a data alignment of 48 selected accessions from GenBank of *Ptilagrostis* species and selected species of 23 other Stipeae genera, with *Phaenosperma globosum* Munro ex Benth. as an outgroup [7, 8]. GenBank accession numbers and voucher information for the materials used in this study are provided in S1 Table.

#### DNA extraction, amplification and sequencing

Total genomic DNA of *Ptilagrostis contracta* was extracted from silica gel-dried leaves collected in the field using a Plant Genomic DNA Kit according to the instructions of the manufacturer (Tiangen Biotech, Beijing, China). ITS sequence data was employed to infer phylogeny in this study. They were amplified and sequenced using primer pairs ITS1 (AAGTCGTAACAAGGTTTCCGTAGGTG) [15] / ITS4 (TCCTCCGCTTATTGATATGC) [16]. The amplification parameters were: initial denaturation phase of 4 min at 94°C; followed by 30 cycles of denaturation at 94°C for 30 s, annealing phase at 55°C–57°C for 30 s, extension phase at 72°C for 1 min 10 s, and final extension at 72°C for 6 min. Automated sequencing was performed by Sino Geno Max Inc. (Beijing, China). Sequences of *P*. *contracta* were deposited at GenBank (S1 Table).

#### Phylogenetic analyses

Sequence data were edited and assembled in the ContigExpress program of the Vector NTI Suite v.6.0 (Informax, North Bethesda, Maryland, U.S.A.). Sequences alignment was done manually using BioEdit v.7.0.5.3 [17]. All gaps were treated as missing data.

Maximum likelihood (ML) analysis was performed using RAxML 7.2.7 [18] from CIPRES cluster web servers [19] with a K80+G model suggested by jModelTest [20] according to the Akaike information criteria [21, 22]. Bootstrap probability (BP) values were calculated for 1000 replicates.

In the Bayesian analysis, the K80+G model was selected by the program jModelTest according to the Bayesian information criteria [23]. Markov chain Monte Carlo (MCMC) iterations with four chains were conducted for 3 000 000 generations, sampling a tree every 1000 generations, with the program MrBayes 3.2.3 [24]. The first 10% trees were discarded as burn-in and the remaining trees were used to determine the posterior probabilities (PP) for branches.

#### Nomenclature

The electronic version of this article in Portable Document Format (PDF) in a work with an ISSN or ISBN will represent a published work according to the International Code of Nomenclature for algae, fungi, and plants, and hence the new name contained in the electronic publication of a PLOS article is effectively published under that Code from the electronic edition alone, so there is no longer any need to provide printed copies.

In addition, the new name contained in this work has been submitted to IPNI, from where it will be made available to the Global Names Index. The IPNI LSIDs can be resolved and the associated information viewed through any standard web browser by appending the LSID contained in this publication to the prefix http://ipni.org/. The online version of this work is archived and available from the following digital repositories: PubMed Central and LOCKSS.

## Results

### Cytology

**Fig 1:**
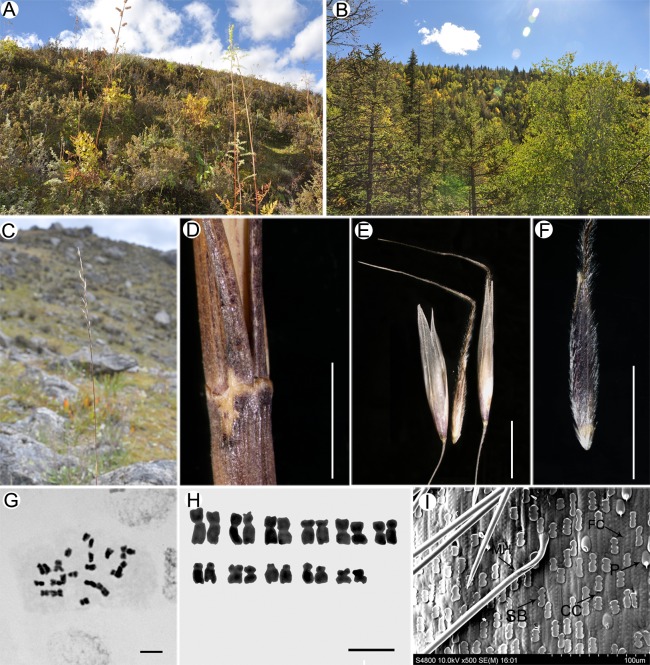
*Ptilagrostis contracta*. A-B. habitat; C. contracted panicle; D. base of panicle branch (pulvinae absent); E. spikelets; F. floret (lemma evenly pubescent); G. chromosomes; H. karyotype; I. Lemma epidermal pattern. Scale bar in D represents 2 mm; in E, F represent 5 mm; in G, H represent 5 μm.

The chromosome number of *Ptilagrostis contracta* is 2n = 22 (Fig 1G), same as the previous observation on other species of *Ptilagrostis* [2, 8]. *Ptilagrostis contracta* has medium and small chromosomes and the length of chromosomes varies from 1.4 μm to 3.6 μm. The karyotype is 2n = 14m+6sm+2st (Fig 1H). Acronyms: m, metacentric chromosome; sm, submetacentric chromosome; st, acrocentric chromosome.

### Lemma epidermal pattern

*Ptilagrostis contracta* has typical *Ptilagrostis* saw-like lemma epidermal pattern (*Ptilagrostis* SLP) [8]. The fundamental cells (FC) are 3–9 times longer than the short cells (SC); side wall of FC sinuous, slightly thickened; SC padded by silica bodies (SB), alternated with cork cell (CC) or not; SB rectangular or oblong with 2–4 shallow contractions; prickles (P) and macro hairs (MH) present (Fig 1I).

### Molecular Systematics

**Fig 2:**
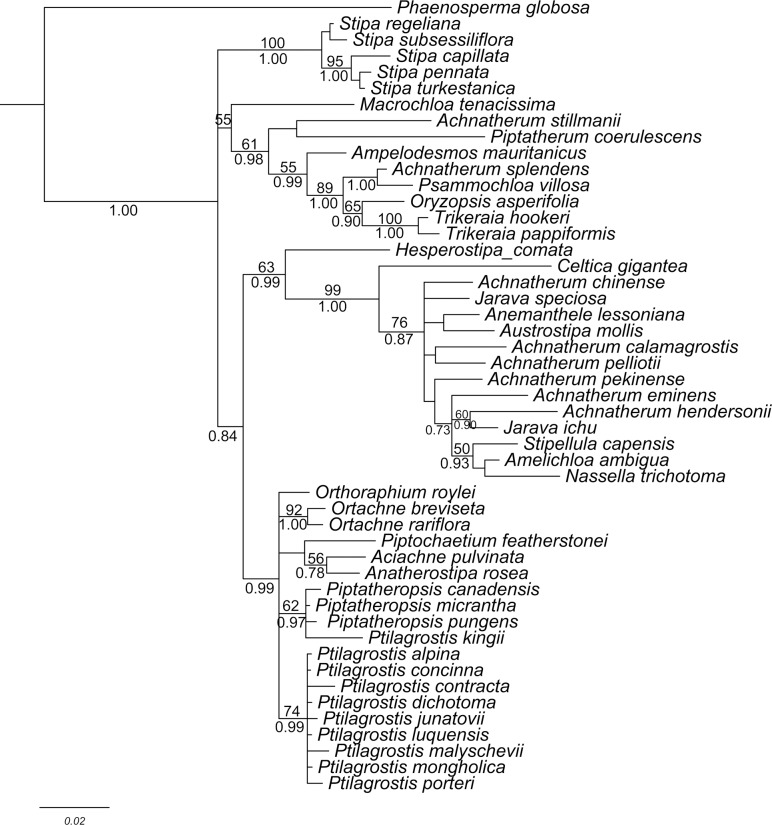
Phylogram of *Ptilagrostis* obtained from MrBayes analysis of ITS dataset. Numbers above branches are support values of ML, numbers below the branches are support values of BI.

BI and ML analyses (Fig 2) produced highly congruent topologies. As shown in Fig 2, several monophyletic clades can be recognized although the internal relationship among the clades are not well-resolved in Stipeae. Monophyletic core *Ptilagrostis* clade including the type of *Ptilagrostis* namely *P*. *mongholica* (Turcz. ex Trin.) Griseb. plus other eight species of *Ptilagrostis* were well supported (74/0.99). However *P*. *kingii* (Bol.) Barkworth was resolved closely related to *Piptatheropsis* Romasch., P.M. Peterson & Soreng. Significantly, the accession of *Ptilagrostis contracta* deeply nested within the core *Ptilagrostis* clade.

### Taxonomic treatment

**Fig 3:**
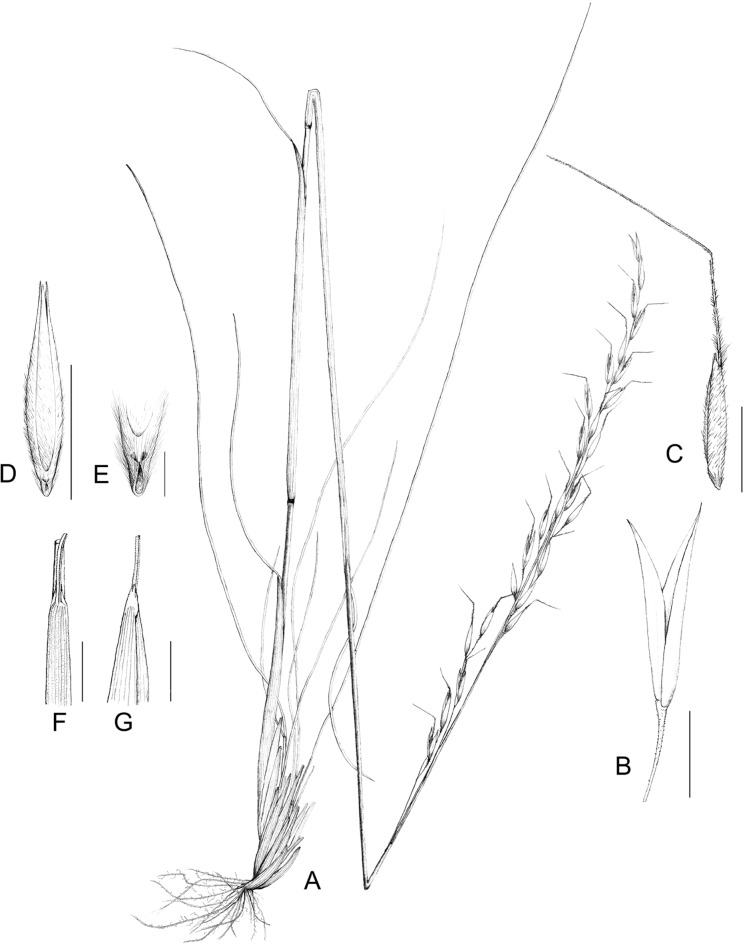
Illustration of *Ptilagrostis contracta*. A. habit; B. glume; C. floret; D. palea; E. callus F. outer surface of ligule; G. inner surface of ligule. Scale bar in B, C, F, G represent 5 mm; in D represent 2 mm; in E represent 0.5 mm (Drawn by Z. S. Zhang).

***Ptilagrostis contracta* Z. S. Zhang & W. L. Chen**, sp. nov. [urn:lsid:ipni.org:names: 77158315–1] (Fig 1 and Fig 3).

**Type:**—CHINA. Sichuan: Litang, 3701 m, 26 September 2014, *Z*. *S*. *Zhang & L*. *L*. *Li 341* (holotype PE!).

#### Diagnosis

*Ptilagrostis contracta* differs from the other species of *Ptilagrostis* in having contracted panicles, 1-geniculate awns with hairy columns and scabrous bristles and evenly pubescent lemmas. In gross morphology, the new species resembles to *P*. *yadongensis* Keng f. & J. S. Tang and *Stipa subsessiliflora* (Rupr.) Roshev. (≡*Ptilagrostis subsessiliflora* (Rupr.) Roshev.) [6, 25, 26] in having contracted panicles. For distinguishing these 3 narrow panicled species, a key is provided here.

### Key to *Ptilagrostis contracta*, *P*. *yadongensis* and *Stipa subsessiliflora*

**Table:** 

1. Awns deciduous, 2-geniculate, hairs in the lower part of the awns 2–3 mm	*S*. *subsessiliflora*
1. Awns persistent,1-geniculate, hairs in the lower part of the awns less than 2 mm	2
2. Plants 8–18 cm tall, upper glumes shorter than the lower glumes by 2 mm, awns plumose throughout	*P*. *yadongensis*
2. Plants 60–105 cm tall, upper glumes subequal to the lower glumes, awns with plumose column and scabrous bristle	*P*. *contracta*

#### Description

Perennial, densely tufted, basal branching intravaginal; culms erect, 60–105 cm tall, ca. 2–3 mm in diameter, 3-noded. Leaf sheaths nearly smooth; leaf blades slender, fold, up to 43 cm, 0.6–1 mm wide, nearly smooth; ligule lanceolate, 1–2.5 mm. Panicles contracted, 13–31 cm long; branches often paired, appressed to the rachis, pulvinae at the base of the branches absent; lower part bare, upper part with few spikelets. Spikelets 10–14 mm long, yellowish-brown or purple at base; glumes subequal, lanceolate-oblong, membranous, hyaline, midrib obvious, lateral veins inconspicuous, lower 10–14 mm long, upper 10–14 mm long; calluses obtuse, ca. 0.5 mm long; lemmas 6–9 mm long, evenly pubescent with hairs ca. 0.5 mm long, apex 2-toothed, teeth 0.3–1 mm long; awns persistent, 1.3–2.0 cm long, 1-geniculate, columns twisted, hairs ca. 0.5–0.7 mm long, bristles scabrous, hairs ca. 0.1 mm long; paleas subequal to lemmas; anthers ca. 2.5 mm long, bearded at apex.

#### Etymology

The epithet contracta refers to the contracted panicles. Chinese name: 紧序细柄茅.

#### Distribution and habitat

**Fig 4:**
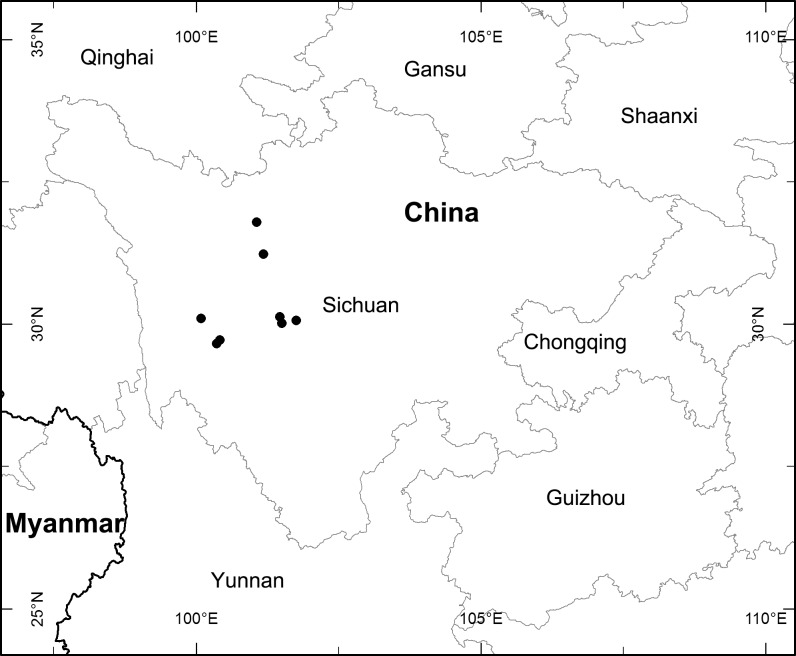
Distribution map of *Ptilagrostis contracta*.

*Ptilagrostis contracta* grows under the birch forests or in the *Rhododendron* thickets at about alt. 3500–4300 m. It is endemic to Qinghai-Tibet Plateau and currently found only in Sichuan province, China (Fig 4).

#### Phenology

Flowering and fruiting from July to October.

#### Conservation Status

*Ptilagrostis contracta* is currently known from 9 locations in Sichuan province within the regions of Dawu, Kangding, Litang and Zamtang. The number of mature individuals is estimated to be ca. 18,000 in the populations. The populations in Kangding and Litang are all close to roads or villages in habitats seriously disturbed by human activities. One population in Kangding (*Z*. *S*. *Zhang & L*. *L*. *Li 375*) possesses no more than 20 mature individuals. The population size of *P*. *contracta* will likely drop to 10,000 in the near future and is close to qualifying for a Vulnerable (VU) species according to the criteria C (IUCN red list categories and criteria, Version 3.1, Second edition) [27]. So *P*. *contracta* was categorized as a Near Threatened (NT) species (A taxon is Near Threatened when it has been evaluated against the criteria but does not qualify for Critically Endangered, Endangered or Vulnerable now, but is close to qualifying for or is likely to qualify for a threatened category in the near future) [27].

#### Additional specimens examined

CHINA. Sichuan: Daocheng Xian & Litang Xian, Hei-tze-shan, 3700 m, 29 August 1934, *C*. *S*. *Liu 1425* (PE); Dawu Xian, 4300 m, 15 September 1974, *Anonymous 5974* (PE); Kangding Xian, 3574 m, 28 September 2014, *Z*. *S*. *Zhang & L*. *L*. *Li 366* (PE); Kangding Xian, 3545 m, 28 September 2014, *Z*. *S*. *Zhang & L*. *L*. *Li 371* (PE); Kangding Xian, 3924 m, 29 September 2014, *Z*. *S*. *Zhang & L*. *L*. *Li 375* (PE); Litang Xian, 3728 m, 24 September 2014, *Z*. *S*. *Zhang & L*. *L*. *Li 336* (PE); Litang Xian, 4100 m, 24 September 1973, *Sichuan Vegetation Survey Team 3987* (PE, N, KUN); Zamtang Xian, 4100 m, 17 July 1975, *Anonymous 9320* (PE).

## Discussion

*Ptilagrostis contracta* is strongly supported within *Ptilagrostis* by our lemma epidermal pattern (LEP) and cytology (2n = 22) data. Molecular evidence from ITS sequence data also support the generic affiliation of *P*. *contracta*. Romaschenko et al [28] reported the incongruence between nuclear and plastid tree in tribe Stipeae, *Ptilagrostis* species formed one clade in ITS tree but separated into two clades in plastid tree. To resolve the phylogentic problem of *Ptilagrostis*, further genetic analyses are needed.

The new species, *Ptilagrostis contracta* is similar to the other species of *Ptilagrostis* by haing intravaginal basal branches, thin leaves, lanceolate ligules, blunt calluses and hyaline glumes but differs by 1-geniculate awns with hairy columns and scabrous bristles and evenly pubescent lemmas which expended the morphological variation of *Ptilagrostis*.

## Supporting Information

S1 Table GenBank accession numbers and voucher information for the materials used in this study. (XLSX)
